# Can Sexual Appeal, Beauty, or Virtue Increase the Opportunity for a Woman to Be Selected as a Spouse? The Mediating Role of Human Uniqueness

**DOI:** 10.3389/fpsyg.2021.698712

**Published:** 2021-09-03

**Authors:** Ji Lai, Daoqun Ding, Xinling Chen, Shenglan Li

**Affiliations:** ^1^Cognition and Human Behavior Key Laboratory of Hunan Province, Hunan Normal University, Changsha, China; ^2^Department of Psychology, Center for Mind and Brain Science, Hunan Normal University, Changsha, China; ^3^Department of Management, Hunan Police Academy, Changsha, China; ^4^Guangdong Key Laboratory of Mental Health and Cognitive Science, School of Psychology, Center for Studies of Psychological Application, South China Normal University, Guangzhou, China; ^5^Normal College, Hunan University of Arts and Science, Changde, China

**Keywords:** women stereotypes, human uniqueness, human nature, mating opportunity, mating values

## Abstract

High mating value is believed to correspond with high mating opportunities. On that premise, this study explores three cues that are linked to women of high long-term mating value, namely a “beautiful” facial appearance, “sexually attractive” body shape, and “virtuous” behavior. With exclusive attention focused on the above cues, this study examines what kind of human attributes would make a contribution to women’s mating opportunities. The results reveal that both “beautiful” women and “virtuous” women were assessed (in this study) as having greater mating opportunities than “sexually attractive” women. In regard to the human attributes, only the “beautiful” woman was assessed as having high levels of human uniqueness and human nature. Meanwhile, “virtuous” women were assessed as having higher levels of human uniqueness but lower levels of human nature. In contrast, “sexually attractive” women were assessed as having lower levels of human uniqueness but higher levels of human nature. In addition, the results of a mediation analysis show that the trait of human uniqueness, and not human nature, was the mediator between the three types of women and women’s mating opportunities. This finding means that, when women have higher levels of human uniqueness, they can acquire more mating opportunities. These findings contribute an improved understanding to why and how “beauty” or “virtue” increases the opportunity for woman to be selected as a spouse.

## Introduction

In human society, mate selection is an important prerequisite for reproduction, providing the first step for individuals who want to enter into marriage and establish families. In the process of choosing a spouse, individuals tend to have certain criteria that they use to define a high-quality mate or a spouse of high mating value. Men focus on three issues: (1) whether the spouse has high reproductive value; (2) whether the spouse’s children have a paternity relationship with the man, and (3) whether the spouse can cooperate with the man to co-nurture their offspring (Buss and Schmitt, 2019). The first issue is associated with women’s physical attractiveness; the second and the third issues are associated with women’s moral qualities.

Female reproductive value is inferred by examining external physical cues (Garza et al., 2016). Women of higher reproductive value show a stronger attractiveness in terms of facial features and having sexual figures (Andrews et al., 2017). These facial features usually include facial adiposity, plump lips, a small chin, thin jaws, and high cheekbones, all of which make the face look attractive (Karremans et al., 2010). Sexual figures are often taken to mean a slender waist, a low waist-to-hip ratio, firm breasts, and a relatively low body mass index (BMI); these features are considered to be reliable indicators of body attractiveness in women (Kościński, 2013; Sugiyama, 2015).

Scientific studies have confirmed that women of high reproductive value usually have the above facial features and sexual figure characteristics. On the one hand, individual differences in facial attractiveness may reflect women’s fertility differences (Jokela, 2009), and even differences in lifespan (Conroy-Beam and Buss, 2019). Studies have found that, at different stages of the menstrual cycle, a woman’s facial attractiveness can change. People’s evaluation of the same woman’s facial attractiveness is higher during her ovulation stage than her luteal phase (Roberts et al., 2004). On the other hand, underweight women (BMI<18.5) or overweight women (BMI>25) are at a higher risk of developing serious diseases such as ovulatory dysfunction (Green et al., 1988) and cardiovascular diseases (Kopelman, 2000). Nonetheless, different subjects and methodological studies have shown that men perceive the facial attractiveness and body shape of potential mates to be important (Buss, 1985; Townsend and Levy, 1990; Bleske-Rechek et al., 2014; Morgan and Kisley, 2014). Furthermore, men are apparently more greatly concerned with women’s body shapes when seeking one-time sexual encounters. However, men pay more attention to women’s facial attractiveness when considering the value of a long-term mate (Morgan and Kisley, 2014).

A woman’s abilities to run a family and raise offspring are inferred by examining her moral qualities and personality traits (Miller, 2007; Buss, 2011). In terms of moral qualities, men are more concerned with female sexual loyalty and controllability (Buss and Schmitt, 1993). Such traits can increase the probability of a biological genetic relationship between men and their future generations (Gil-Burmann et al., 2002). In terms of personality traits, men value characteristics such as kindness, reliability, and congeniality (Apostolou, 2015), which are considered to be essential to creating a warm and healthy family life atmosphere.

A survey about marriage and love was conducted in China. The results revealed that a female’s physical (e.g., having a small chin), behavioral (e.g., chastity), and personality traits (e.g., being honest, kind, faithful, and sexually modest) are the values most favored by men, largely because having women with these characteristics as spouses or partners would relieve the men of any paternity uncertainty (Chang et al., 2010). In addition, traditional Chinese cultures provide guidelines for women with regard to being “good wives and mothers.” These traditions require that a woman should be loyal to her husband and take care of every family member, including their parents-in-law. The woman should also deal with family affairs diligently and ensure the children are educated (Chang et al., 2010; Schlomer et al., 2011). Men’s potential long-term mates should have high attractiveness in terms of facial appearance, good personalities, and the ability to manage housework. With those traits, women can give their husbands enough care and support in the couple’s future married life (Yan et al., 2018). Since the implementation of the policy of economic reform and opening-up in China, women’s social status has significantly improved, with increased education and independence. This, then, has caused an improvement in terms of women’s knowledge and ability, and more women have achieved economic independence. As a result, women’s abilities to support their families have been increasingly enhanced, and those abilities are becoming increasingly valued by men. Men also prefer mates with such attributes as hard work and generosity, because those attributes also signal a woman’s potential ability to support her family (Hao, 2011; Lu et al., 2015).

High mating values are believed to correspond with high mating opportunities. Therefore, to increase their mating opportunities, women adjust their behaviors to accommodate men’s abovementioned preferences. For example, women like to put on makeup when dating, because they believe makeup makes them look healthier (Bielfeldt et al., 2013; Jones et al., 2016) by reducing the signs of age and increasing facial attractiveness (Porcheron et al., 2013; Jones et al., 2015). Women will spend more money on beauty products to increase their attractiveness despite economic difficulties. This behavior is viewed as an instinctive response to the intensified competition for spouses in difficult times (Hill et al., 2012). Furthermore, for reasons of mating motivation, women are willing to help others in public (Griskevicius et al., 2007). In other words, women want to be seen in public to be kind, helpful, moral, and generous as this type of behavior could be admired by members of the opposite sex, thus enhancing the women’s mating value. Therefore, women are very familiar with men’s mate preferences. In fact, women have similar views regarding a female’s mating value as men.

Although having a sexually attractive body (e.g., a slender waist and firm breasts), beautiful facial appearance (e.g., plump lips and a small chin), and the qualities associated with being “good wives and mothers” (e.g., kindness, diligence, and love) are the important mating values, focusing exclusively on such mating values could cause a cognitive bias against women, incurring certain negative stereotypes (Cikara et al., 2011). For example, women in hot clothes attract others’ attention to their sexually appealing bodies (e.g., firm breasts and warped fruity buttocks). However, dressing this way could also incur being labeled with negative stereotypes such as having lower moral value (e.g., engage in extramarital affairs) and lesser abilities (Loughnan et al., 2010). Also, women with beautiful facial appearance, or those who are admired for their looks, could equally incur being labeled with such negative stereotypes as lacking vitality and warmth (Heflick and Goldenberg, 2009) or having a lesser ability to experience pain (Philippe et al., 2018). How, exactly, can these stereotypes be incurred? Haslam’s two-dimensional model of humanness may shed some light.

According to Haslam’s two-dimensional model, human uniqueness and human nature constitute the two senses (or dimensions) of humanness (Haslam, 2006). Human uniqueness literally means unique human characteristics. These characteristics, in turn, such as morality (e.g., being well-educated, having secondary emotion such as regret and grateful), maturity (e.g., being socialized, civilized, literate), and rationality (e.g., self-control, behavior driven by reason) represent the cultural layer of humanness. These are the characteristics that separate humans from animals, and such features are acquired through cultivation. Human nature, as the second dimension, is comprised of the innate and essential part of one’s humanness. The features are defined by vitality, emotions (e.g., happiness, anger, fear, and other primary emotions), cognitive openness (e.g., curiosity and flexibility), and agency (e.g., taking the initiative to do things on one’s own). These are the features that separate humans from objects or automata; such characteristics should also be the same in every person, regardless of the individual’s cultural background (Haslam, 2006; Haslam and Loughnan, 2014).

The stereotypes that stem from focusing solely on women’s sexually appealing bodies lead to men viewing women as individuals with a lesser degree of humanness. This view also has detrimental effects on the treatment of women, including an increase in and the facilitation of aggression (Haslam, 2006; Haslam and Loughnan, 2014), rape proclivity (Cikara et al., 2011; Rudman and Mescher, 2012), and deception (Rudman and Mescher, 2012; Xiao et al., 2017). Moreover, recent findings show that the stereotypes that stem from focusing solely on beautiful facial appearance are also correlated with the perception that a woman is less able to feel pain. Such a perception could facilitate aggression toward women (Morris et al., 2018). Therefore, examining whether these negative stereotypes can also influence women’s mating opportunities is very worthwhile.

To date, some evidence has linked the stereotypes of “beautiful” women and “sexually attractive” women with devalued humanness, but more empirical studies are still required, in order to validate those findings. In addition, the relationships between humanness and women’s mating opportunities have not been tested. Furthermore, whether the two independent dimensions of humanness – human uniqueness and human nature –can make similar contributions to mating opportunities is still under exploration. It is important to note that no direct research exists that explores what kind of humanness can be derived from the stereotypes of “virtuous” personalities represented by “good wives and mothers.” However, “virtuous” personalities have traditionally been considered to be important in Chinese culture when judging women’s mating value. This study examines the effects of stereotypes on the perceived women’s mating opportunities by manipulating the three kinds of stereotypes represented by “sexually attractive” women, “beautiful” women, and “virtuous” women, respectively. More specifically, three kinds of stereotypes were induced by attracting participants to focus on the features of a sexually appealing body, beautiful facial appearance, and “virtuous” behavior. Checking the effects of human uniqueness and human nature is important if one is to understand the relationships of perceived women’s mating opportunities and their stereotypes. As mentioned above, women may have the same consciousness as men when judging another female’s mating value, and as there is no evidence indicating any gender difference when judging the humanness of women, this study will further testify to the gender effect in these aspects.

## Materials and Methods

### Participants

A power analysis, conducted in G^*^power (Version 3.1.9.2; Faul et al., 2007), indicated that a minimum total sample size of *N*=211 was required to achieve sufficient power (1–*β*=0.80) with a medium effect size of *f*=0.25. A total of 251 undergraduate participants were recruited through a psychological health education course; all participated in this study through the Wenjuanxing questionnaire platform,^1^ and everyone who participated was paid five CNY. Three questionnaires were excluded from the final results, because three individuals reported their sexual orientation as being homosexual or bisexual. Another eight questionnaires were excluded because they failed to pass the attention test, which stated, “This question is an attention test, please choose number 2.” Those eight respondents did not choose the number 2. In the “sexually attractive” category, the sample consisted of 39 males (*M*_age_=18.79years, *SD*_age_=0.83) and 36 females (*M*_age_=21.83years, *SD*_age_=17.50). In the “beautiful” category, the sample consisted of 37 males (*M*_age_=18.95years, *SD*_age_=1.60) and 42 females (*M*_age_=19.00years, *SD*_age_=2.34). In the “virtuous” category, the sample consisted of 44 males (*M*_age_=18.89years, *SD*_age_=0.92) and 42 females (*M*_age_=18.64years, *SD*_age_=1.03). The three categories had no differences in gender, *χ*^2^ (1, *N*=240)=0.48, *p*=0.79. No main effect of gender was noted on age, *F*(1, 234)=1.13, *p*=0.289, $\;\eta_{P}^{2}$=0.005. No main effect of categories was noted on age, *F*(2, 234)=1.16, *p*=0.317, $\;\eta_{P}^{2}$=0.010, and no interaction of gender and category was noted on age, *F*(2, 234)=1.34, *p*=0.263, $\;\eta_{P}^{2}$=0.011. This study was approved by the Research Ethics Committee of Hunan Normal University. Informed consent was obtained from all the participants who involved in this study.

### Materials

#### Image Prime

Participants were asked to perform an impression task, and they were presented with one profile consisting of four images. Three profiles were used in total, depicting “sexually attractive” women, “beautiful” women, and “virtuous” women, respectively. In the “sexually attractive” category, the woman wears a sexy black mini skirt with bare skin above her chest and below her thighs. Under illumination, she swayed her body, highlighting her firm breasts and hips. In the “beautiful” category, the woman dressed fashionably but in non-revealing clothing; her face appeared glamorous and polished. In the “virtuous” category, the woman wore simple clothes to do some things, such as making pottery pots, taking photographs, choosing books, and hugging dolls. These actions were intended to show the characteristics of ingenuity, warmth, and an orchid heart. This study used three different women for each category. The priming pictures are available at https://doi.org/10.17605/osf.io/qr7jv.

To determine the appropriateness of the images, pilot testing was conducted on the Wenjuanxing questionnaire platform (*n*=95). In all, 27 undergraduate participants (21 females, *M*_age_=24.52years, *SD*_age_=7.20 and 6 males, *M*_age_=22.67years, *SD*_age_=2.07) viewed the images of the sexually attractive woman, 32 undergraduate participants (17 females, *M*_age_=18.35years, *SD*_age_=1.50 and 15 males, *M*_age_=20.13years, *SD*_age_=2.17) viewed the images of the beautiful woman, and 36 undergraduate participants (18 females, *M*_age_=18.06years, *SD*_age_=0.54 and 18 males, *M*_age_=18.67years, *SD*_age_=2.79) viewed the images of the virtuous woman. Then, participants were asked to respond to questions that were designed to assess the perceived sexual attractiveness (“How sexually attractive is this woman?”), the value of a beautiful face (“How beautiful is this woman?”), and the value of internal attributes (“How important is this woman’s virtuous personality?”). Each question was rated from 1, for not at all to 7, for very much. There was a significant effect of the prime on perceived sexual attractiveness: *F*(2, 92)=9.72, *p*<0.001, $\eta_{P}^{2}$=0.18. The woman in the sexually attractive category was rated as more sexual than both the beautiful woman and the virtuous woman (*p*_s_<0.004); the latter two did not differ from one another in their ratings (*p*=0.080). A significant effect of the prime on perceived face value was also noted: *F*(2, 92)=5.47, *p*=0.006, $\eta_{P}^{2}$=0.11. The beautiful woman was assigned a higher value for her face than both the sexually attractive woman and the virtuous woman (*p*_s_<0.032); the latter two did not differ from one another in their scores (*p*=0.62). Furthermore, a significant effect of the prime on perceptions of internal attributes was noted: *F*(2, 92)=20.31, *p*<0.001, $\eta_{P}^{2}$=0.31. The virtuous woman was valued more highly for her virtuous trait than either the beautiful woman or the sexually attractive woman (*p*_s_<0.021). Meanwhile, the beautiful woman was rated as having a higher value for her virtuous trait than the sexually attractive woman (*p*<0.001). In addition, participant gender did not interact with the prime to affect results for any outcome (*p*_s_>0.48).

#### Humanness

Participants were assigned to complete both the human uniqueness (i.e., humble, trustworthy, analytical, helpful, sincere, polite, civilized, conservative, thorough, competent, tolerant, and refined) and human nature subscales (i.e., fun-loving, sociable, active, passionate, emotional, talkative, friendly, imaginative, ambitious, artistic, curious, and impulsive). Participants were asked to determine the extent of the 24 typical traits of the woman in the profile (from 1, meaning very atypical, to 5, meaning very typical). The 24 traits were adopted from previous research (Haslam et al., 2005; Heflick et al., 2011; Noël et al., 2021), and participants were asked to rank the women in terms of each of the traits. Composite scales were constructed by averaging target trait ratings on the items which were identified as representing the domains of human uniqueness (*α*=0.94) and human nature (*α*=0.88).

#### Possibility of Being Married

Participants were asked to indicate the possibility of each of the three women stereotypes being chosen as a wife. Responses were recorded on a 7-point scale, ranging from 1 (no possibility of being chosen) to 7 (certain to be chosen).

#### Demographics

Participants completed a short demographic questionnaire to determine their age, gender, and sexual orientation.

## Results

### Humanness Ratings

A repeated-measures ANOVA was conducted, with 2 (participant gender: female vs. male)×3 (stereotypes: “sexually attractive” woman vs. “beautiful” woman vs. “virtuous” woman)×2 (humanness dimension: human uniqueness vs. human nature). Humanness is a within-subjects variable, while participant gender and the stereotypes are between-subjects variables. The ratings of human uniqueness and human nature were standardized, so that the differences between the two could be compared. The results show the main effect of humanness is not as significant (*p*=0.55) as the main effect of participant gender (*p*=0.92). On the contrary, the stereotypes had the significant main effect, *F*(2,234)=7.41, *p*=0.001, $\eta_{P}^{2}$=0.60. The pairwise comparison also revealed that the “sexually attractive” woman (*M*=−0.30, *SD*=0.83) was seen to have lower humanness than the “beautiful” woman (*M*=0.12, *SD*=0.84), *p*=0.006. The “sexually attractive” woman was also seen to have lower humanness than the “virtuous” woman (*M*=0.16, *SD*=0.83), *p*=0.001, but no difference was found between the “beautiful” woman and the “virtuous” woman (*p*=1.00). The interaction effect between humanness and participant gender was also not significant (*p*=0.63).Interestingly, a significant interaction effect between humanness and stereotypes was observed: *F*(2,234)=50.93, *p*<0.001, $\eta_{P}^{2}$=0.30. A follow-up simple effects analysis showed the following: The human nature rating was significantly higher than the human uniqueness rating in the “sexually attractive” woman image prime conditions: *F*(1, 234)=61.98, *p*<0.001, $\eta_{P}^{2}$=0.21. However, the human nature rating was significantly lower than the human uniqueness rating in the “virtuous” woman image prime conditions: *F*(1,234)=38.68, *p*<0.001, $\eta_{P}^{2}$=0.14. Furthermore, no significant difference exists between the human nature rating and the human uniqueness rating in the “beautiful” woman image prime conditions: *p*=0.28. In addition, the interaction effect between humanness, stereotypes, and participant gender was not observed, *p*=0.48 (for more details, see Table 1).

**Table 1:** Descriptive statistics of humanness and the possibility of being married M(SD).

Stereotypes	Human uniqueness				Human nature				Possibility of being married			
	*Male*	*Female*	*Total*	*P(M-F)*	*Male*	*Female*	*Total*	*P(M-F)*	*Male*	*Female*	*Total*	*P(M-F)*
“Sexually attractive” woman	−0.52 (0.99)	−0.86 (1.09)	−0.69 (0.10)	0.097	0.20 (0.72)	−0.033 (1.35)	0.085 (0.12)	0.31	3.74 (1.57)	4.11 (1.72)	3.92 (1.64)	0.25
“Beautiful”woman	0.20 (0.77)	0.14 (0.72)	0.17 (0.098)	0.76	0.21 (0.76)	−0.081 (0.98)	0.064 (0.11)	0.19	4.62 (1.26)	4.57 (1.35)	4.59 (1.30)	0.87
“Virtuous” woman	0.23 (1.00)	0.67 (0.56)	0.45 (0.094)	0.019^*^	−0.33 (1.23)	0.082 (0.73)	−0.12 (0.11)	0.056	4.00 (1.38)	5.10 (0.96)	4.53 (1.31)	< 0.001^***^

*M-F refers to the gender difference comparison*;

* *p< 0.05*;

^**^p< 0.01;

*** *p<0.001*.

### Ratings of Possibility of Being Married

To test the differences in the possibility of women being married within each category, an ANOVA was conducted, using participant gender (female and male) and the three stereotypes (“sexually attractive” woman, “beautiful” woman, and “virtuous” woman). In this study, the main effect of gender is significant: *F*(1, 234)=6.92, *p*=0.009, $\eta_{P}^{2}$=0.029. Females (*M*=4.59, *SD*=1.42) rated higher than males (*M*=4.12, *SD*=1.42) when considering the opportunities that women have to be married. The main effect of stereotypes is also significant: *F*(2, 234)=5.60, *p*=0.004, $\eta_{P}^{2}$=0.046. The “sexually attractive” woman received lower rating scores than the “beautiful” woman (*p*=0.003); the “sexually attractive” woman also rated lower than the “virtuous” woman when considering the opportunities that women have to be married (*p*=0.005). However, no significant difference was noted between the “beautiful” woman and the “virtuous” woman (*p*=0.82). The interaction between gender and stereotypes was significant: *F*(2, 234)=3.63, *p*=0.028, $\eta_{P}^{2}$=0.030. For males, the “beautiful” woman was rated as having a better chance of being married than the “sexually attractive” woman: *p*=0.006. The “beautiful” woman was also rated as having a better chance of being married than the “virtuous” woman: *p*=0.045. However, no significant difference was noted between the “sexually attractive” woman and the “virtuous” woman, *p*=0.40. For females, the “virtuous” woman was rated as having a better chance of being married than the “sexually attractive” woman: *p*=0.002. No difference was noted between the “virtuous” woman and the “beautiful” woman: *p*=0.084. Also, no difference was found between the “sexually attractive” woman and the “beautiful” woman: *p*=0.14. These findings indicate that male participants rated “pretty” women as having a better chance of being married, while women tend to believe that men will be more likely to choose “virtuous” women as partners (for more details, see Table 1).

### Mediation Analysis

To test whether the various ways of categorizing women could lead to different impacts on their marriage opportunities (based on humanness ratings), a mediation analysis was conducted through a bootstrapping approach. Effects were derived from 5,000 bias-corrected bootstrap samples, with 95% CI. The image prime involved three categories (the “sexually attractive” woman, the “beautiful” woman, and the “virtuous” woman) and was dummy coded, with the “virtuous” woman category as the reference category (D1: 0=“virtuous” woman, 1=“sexually attractive” woman; D2: 0=“virtuous” woman, 1=beautiful woman). The model assesses the effect of one dummy-coded variable by controlling for the other. The human uniqueness and human nature trait composites were included as mediators operating in parallel. The hypothesized model fit the data well: *χ*^2^/*df*=32.60, CFI=1.000, TLI=1.000, RMSEA=0.000.

### Direct Effects

In the first stage of the model, the effect of the three kinds of stereotypes on humanness outcomes was examined. The “beautiful” woman was perceived as having fewer human uniqueness traits than either the “virtuous” woman [*b*=−0.13, *SE*=0.059, *p*=0.027, *CI* (−0.24, −0.009)] or the “sexually attractive” woman [*b*=−0.52, *SE*=0.070, *p*<0.001, *CI* (−0.65, −0.38)]. Conversely, the “beautiful” woman did not receive significantly higher scores than the “virtuous” woman for human nature traits (*p*=0.22). The “sexually attractive” woman was also not rated significantly higher than the “virtuous” woman on human nature traits (*p*=0.20).

The direct effect of the stereotypes on the possibility of being married was not significant for the “beautiful” woman category (*p*=0.14). This finding indicates that “beautiful” women are considered to be as likely as “virtuous” women to be married. When the “sexually attractive” woman is compared with the “virtuous” woman, the effect on the possibility of being married is not significant (*p*=0.29). This finding suggests that the “sexually attractive” woman was not perceived as being less likely than the “virtuous” woman to be married.

### Indirect Effects

**Figure 1:**
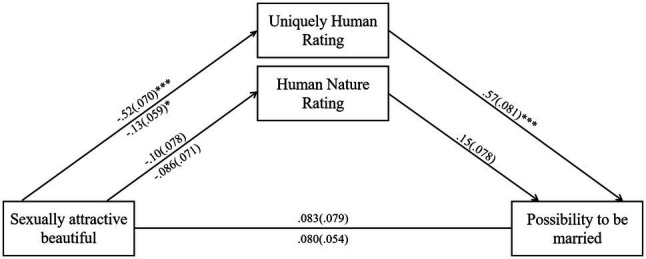
Path estimates for the mediation effect of the “sexually attractive” woman and “beautiful” woman image prime on the possibility of being married through human nature and uniquely human rantings. For paths at the first stage of the model, values above the line reflect estimates for the “sexually attractive” woman image, relative to the control. Values below the line reflect estimates for the “beautiful” woman image, relative to the control (coded 0=virtuous woman; 1=comparison). Also, ^*^p<0.05; ^**^p<0.01; and ^***^p<0.001.

Based on the participants’ perceptions of human uniqueness, the relative indirect (mediation) effect of women’s stereotypes on the possibility of being married was significant for both the “beautiful” woman [effect=−0.073, *SE*=0.036, *CI* (−0.15, −0.01)] and the “sexually attractive” woman [effect=−0.30, *SE*=0.062, *CI* (−0.42, −0.19)]. Compared to the “virtuous” woman, the “sexually attractive” woman and the “beautiful” woman were both attributed fewer human uniqueness traits; this reflects a lower possibility that these women would be selected for marriage. In addition, the relative indirect effect of human nature ratings on the possibility of being married was not significant for either the “sexually attractive” woman or the “beautiful” woman, compared to the “virtuous” woman. Figure 1 presents the full path model; Table 2 displays the estimates of the relative indirect effects.

**Table 2:** Relative indirect effects.

			95% CI	
Mediator	Effect	SE	LL	UL
**Uniquely human rating**				
Sexually attractive vs. virtuous	−0.30	0.062	−0.42	−0.19
Beautiful vs. virtuous	−0.073	0.036	−0.15	−0.01
**Human nature rating**				
Sexually attractive vs. virtuous	0.015	0.016	−0.005	0.06
Beautiful vs. virtuous	0.01	0.014	−0.005	0.054

*Statistical significance is inferred from confidence intervals that do not contain zero; CI, confidence interval; LL, lower limit; UL, upper limit*.

Additional analyses were conducted to determine whether participant gender moderated these effects. No direct effects of gender on the outcome were noted (*p*_s_>0.072).

## Discussion

This study explores three cues linked to women with high long-term mating value, namely a “beautiful” facial appearance, a “sexually attractive” body shape, and “virtuous” behaviors. With exclusive attention being paid to the above cues, participants were asked to assess the human uniqueness and human nature of women, as well as the women’s mating opportunities. This study found that a low assessment of women’s human uniqueness could negatively affect women’s long-term mating opportunities. Interestingly, this result was found in both male and female participants.

Speculation about the relationship between humanness and mating opportunities was confirmed by this study’s experimental work. What is surprising about the relationship is that only women’s human uniqueness has an effect on women’s mating opportunities. Human uniqueness is acquired through cultivation and is thus strongly influenced by cultural backgrounds. In contrast, human nature is a characteristic that represents an individual’s inner desires, which occur regardless of the culture or background. Our traditional Confucian Culture emphasizes the view of “preserving the heaven and destroying human desires.” This culture advocates the unity of reason and desire, to practice reasonable abstinence, and that human beings can be reformed through education. This trend of thoughts values human uniqueness more than human nature and has obviously provided guidelines regarding individuals’ judgments.

With regard to women’s mating opportunities, both stereotypes of “beautiful” women and “virtuous” women acquired higher mating opportunity ratings than “sexually attractive” women. The reason why “sexually attractive” women have lower mating opportunities is that these women are assumed to have lower moral value, and just as stated in the previous section, low moral values goes against the spirits of traditional culture. Meanwhile, both male and female participants considered that the “beautiful” woman had the advantage in terms of getting mating opportunities. Actually, beautiful women are more likely than average-looking women to be pursued by members of the opposite sex (Morgan and Kisley, 2014). The results of this study are consistent with that finding. Further, there were gender differences when judging women’s mating opportunities, in that female participants assessed more mating opportunities than males, especially in the category of “virtuous” women. These gender differences could be explained by in-group preference, which means that people could be more optimistic to an in-group member (e.g., when females were assessing the women stereotypes in our study) than to an out-group member (e.g., when the males were assessing the women stereotypes in our study). With regard to “virtuous” women, the reason for the conservative estimation of mating opportunities made by males in this study is that “virtuous” women failed to meet the criteria of having the ability to support a family. The rules of traditional culture are believed to suggest that it is virtuous for women to stay home and obey others’ orders (Ma et al., 2008; Tianyu, 2016; Aihong, 2018). Since the implementation of the policy of economic reform and opening up, more women have achieved economic independence. As a result, women’s abilities to support their families have been increasingly valued.

With regard to humanness, both “beautiful” women and “virtuous” women were assessed as having higher levels of humanness than “sexually attractive” women. Further, in this study, humanness was divided into two sub-dimensions, human uniqueness and human nature. When the two sub-dimensions were tested, only the “beautiful” woman was assessed as having high levels of human uniqueness and human nature. This result means that focusing exclusively on facial appearance may not only evoke a negative stereotype, but conversely, could also result in positive impressions. Affected by the “beauty is good” effect, people seem to believe that beautiful individuals have all the positive qualities, such as sociability, affinity, understanding, and a lively personality, as well as ability (Dion et al., 1972; Zebrowitz and Rhodes, 2004). Meanwhile, the “virtuous” woman was assessed in this study as having higher human uniqueness but lower human nature. In contrast, the “sexually attractive” woman was assessed as being lower in human uniqueness but higher in human nature. These findings suggest that “virtuous” women suppress their inner desires and behave in a way that is in accordance with cultural approval. In contrast, “sexually attractive” women’s behavior is in accordance with their hearts’ desires; the behavior is not done deliberately (Vaes et al., 2010).

In this study, the theory of the two-dimensional mode of humanness is extended, from the area of aggression to the area of mating opportunities. The findings of this research verify the mating theories of evolution psychology (Sprecher et al., 1994; Berry and Miller, 2001). Importantly, this study offers another perspective for understanding mating opportunities by highlighting the role of humanness. Previous researches attempted to interpret the mating phenomenon from the theory of the “good genes” hypothesis or the “big five” personality model (Malouff et al., 2010; Walker and Vetter, 2016).

Another key innovation in this study is the exploration of the stereotype of “virtuous” women. In traditional culture, the image of the “virtuous woman” provides guidelines about moral standards and principles to regulate women’s behavior. This tradition has encouraged men to develop a negative prejudice toward such women because men were led to believe such women were pedantic, inflexible, and depressed. However, there is scarce knowledge that would help with understanding the housewife in Western culture. Future studies are needed to identify and discuss similarities in this particular image of women between Chinese and Western cultures.

## Data Availability Statement

The raw data supporting the conclusions of this article will be made available by the authors, without undue reservation.

## Ethics Statement

The studies involving human participants were reviewed and approved by the Ethics Committee of Hunan Normal University. The patients/participants provided their written informed consent to participate in this study.

## Author Contributions

JL and DD designed the research and wrote the manuscript. JL and XC performed the research. DD, JL, and LL analyzed the data and were involved in the interpretation of data. All authors contributed to the article and approved the submitted version.

## Conflict of Interest

The authors declare that the research was conducted in the absence of any commercial or financial relationships that could be construed as a potential conflict of interest.

## Publisher’s Note

All claims expressed in this article are solely those of the authors and do not necessarily represent those of their affiliated organizations, or those of the publisher, the editors and the reviewers. Any product that may be evaluated in this article, or claim that may be made by its manufacturer, is not guaranteed or endorsed by the publisher.

## Footnotes

^1^ https://www.wjx.cn/
